# Fast selective homogeneous extraction of UO_2_^2+^ with carboxyl-functionalised task-specific ionic liquids

**DOI:** 10.1038/srep44100

**Published:** 2017-03-14

**Authors:** Yinyong Ao, Jian Chen, Min Xu, Jing Peng, Wei Huang, Jiuqiang Li, Maolin Zhai

**Affiliations:** 1 Institute of Nuclear Physics and Chemistry, China Academy of Engineering Physics, Mianyang 621900, P. R. China; 2 Beijing National Laboratory for Molecular Sciences (BNLMS), Department of Applied Chemistry, College of Chemistry and Molecular Engineering, Peking University, Beijing 100871, P. R. China

## Abstract

The carboxyl-functionalised task-specific ionic liquid of 1-carboxymethyl-3-methylimidazolium bis(trifluoromethyl-sulfonyl)imide ([HOOCmim][NTf_2_]) was used as solvent and extractant for UO_2_^2+^ extraction from aqueous solution. A homogeneous phase of [HOOCmim][NTf_2_]-H_2_O system could be achieved at 75 °C, and 86.8 ± 4.8% of UO_2_^2+^ was separated from the aqueous solution after vibrating for only 1 min. Furthermore, nearly 97.3 ± 2.9% of UO_2_^2+^ was stripped from [HOOCmim][NTf_2_] phase by 1 M HNO_3_ solution. K^+^, Na^+^, Mg^2+^, Dy^3+^, La^3+^, and Eu^3+^ have little influence on the homogeneous extraction of UO_2_^2+^, and the extraction efficiency of UO_2_^2+^ still remained at *ca*. 80%. Experimental and theoretical study on the selectivity of [HOOCmim][NTf_2_]-H_2_O system were performed for the first time. Density functional theory calculation indicates that the solvent effect plays a significant role on the selectivity of [HOOCmim][NTf_2_]-H_2_O.

Room-temperature ionic liquids (RTILs) are liquid salts at or around room temperature. In recent years, RTILs have received increasing attention because of their unique physicochemical properties, such as negligible vapour pressure and strong ability to solubilise metal complexes^1^,^2^,^3^,^4^. They have potential as solvents for separation of metal ions from ores^5^,^6^,^7^. To date, most RTILs have only been used as diluents during liquid-liquid extraction^8^,^9^,^10^,^11^,^12^,^13^,^14^,^15^,^16^. Various types of functionalised task-specific ionic liquids (TSILs) have been designed to improve the properties of ionic liquids^17^,^18^,^19^,^20^. The presence of functional groups in either the cation or anion of these ionic liquids allows them to be used both as solvent and extractant in solvent extraction systems without additional extractant. The solubility of TSILs in water can be adjusted by incorporating functional groups into the ionic liquids, enabling creation of temperature-sensitive TSILs. A two-phase TSILs-H_2_O mixture can be converted to one homogeneous phase by raising temperature, and the two-phase equilibrium can be re-established by reducing temperature^21^,^22^,^23^. The long equilibration time for extraction can be greatly reduced by formation of a homogeneous phase.

There is an urgent need for rapid extraction of U(VI) species for separation of uranium from ores in the nuclear fuel cycle^10^,^19^, and there have been many publications on the extraction of U(VI) species^6^,^24^,^25^,^26^,^27^. The fast, selective separation of U(VI) species is of great interest for applications in the nuclear fuel cycle, and has been the subject of several theoretical and experimental studies^10^,^24^. Most studies have proposed methods requiring an extractant in the RTILs phase for selective extraction of U(VI)^28^,^29^,^30^,^31^. Unfortunately, traditional extraction processes commonly need a long equilibration time, which limits their practical application. In addition, RTILs are only used as diluents in these processes, while the required additional extractant. Hoogerstraete *et al*. designed a homogenous extraction system by using binary mixtures of betainium bis(trifluoromethylsulfonyl)imide ionic liquid and H_2_O^17^,^32^. This system showed that effective extraction of trivalent rare-earth, indium, gallium, neodymium ions^18^, and uranyl species^33^ can be achieved by homogeneous extraction without additional any extractant. Homogeneous liquid–liquid extraction of neodymium(III) has also been achieved using choline hexafluoroacetylacetonate in the ionic liquid choline bis(trifluoromethylsulfonyl) imide^34^. Recently, Dupont *et al*. used a functionalised ionic liquid for the selective dissolution and revalorization of Y_2_O_3_:Eu^3+^ from lamp phosphor waste^35^. Nockemann *et al*.^19^ found that U(VI) oxide could be dissolved in three different ionic liquids functionalised with a carboxyl group, and three carboxyl groups coordinated bidentately to the uranyl species in the crystal structure of U(VI)-TSILs complexes. Sasaki *et al*.^33^ reported that the extractability of UO_2_^2+^ at near 62% was achieved by using betainium bis(trifluoromethylsulfonyl)imide ionic liquids. Fast selective homogeneous extraction of U(VI) species from lanthanides by TSILs without addition of any extractant will be of great significance in the nuclear fuel cycle.

Herein, a new fast homogeneous extraction system using 1-carboxymethyl-3-methylimidazolium bis(trifluoromethyl-sulfonyl)imide ([HOOCmim][NTf_2_], Fig. 1) both as diluent and extractant has been designed. Fast homogeneous extraction and traditional liquid-liquid extraction for the removal of UO_2_^2+^ were separately studied in this work, and the selectivity of [HOOCmim][NTf_2_] and the influence of metal ions on the extraction of UO_2_^2+^ were also carefully assessed. Furthermore, a theoretical study was conducted on the selectivity of [HOOCmim][NTf_2_].

The [HOOCmim][NTf_2_]-H_2_O system forms a homogeneous phase when the temperature is increased to 75 °C (Fig. 2), and two-phase equilibrium can be re-established by reducing the temperature. Accordingly, the phase-transition behaviour of the [HOOCmim][NTf_2_]-H_2_O mixture was used to remove UO_2_^2+^ from the aqueous phase. The two-phase [HOOCmim][NTf_2_]-H_2_O mixture was kept at a constant temperature of 75 °C for 10 min and then homogenised by a vibrating mixer for 1 min. Extraction efficiency (*E*_U_) of 86.8 ± 4.8% for UO_2_^2+^ was obtained at 60 °C, and 83.2 ± 4.0% efficiency was achieved after cooling to 30 °C. Treatment of cooling to 30 °C was chosen in the following extraction process. The phase behaviour of the [HOOCmim][NTf_2_]-H_2_O mixture is of great importance for the design of extraction experiments, so the percentage rate ([R]) of [HOOCmim]^+^ from organic phase to aqueous phase was studied first. The [R] of [HOOCmim]^+^ from organic phase to aqueous phase are calculated based on the solubility ([**S**]) of [HOOCmim]^+^ in water and the mass of aqueous phase after equilibrium. The [R] is calculated as follows: [R] = m_aq_ × ([**S**]/(1 + [**S**]))/m_TSILs_ × 100%, where *m*_aq_ is the mass of aqueous phase after equilibrium. *m*_TSILs_ is the initial mass of [HOOCmim][NTf_2_].

As shown in Fig. S1, the [R] of [HOOCmim]^+^ decreased with the increase of phase ratio V_RTILs_/V_H2O_, where V_TSILs_ and V_H2O_ represent the initial volumes of [HOOCmim][NTf_2_] and water, respectively. When V_RTILs_/V_H2O_ = 1, the solubility of [HOOCmim]^+^ in water was about 4.7 ± 0.1% (Figs S2 and S3). The [R] of [HOOCmim]^+^ was calculated at near 7.5 ± 0.1% and changed slightly as the equilibration time increased (Fig. S3), indicating that the [HOOCmim][NTf_2_]-H_2_O system can maintain a low [R] at V_RTILs_/V_H2O_ = 1. For the purpose of comparative analysis, the solubility of [HOOCmim]^+^ in 1.0 HNO_3_ was determined to be approximately 6.3 ± 0.1%, which is higher than that in water. A general chemical cation exchange model, involving a combination of the H^+^ and cationic species from an acidified aqueous phase toward an ionic liquid phase, was proposed by Billard *et al*.^11^. Accordingly, the solubility of [HOOCmim]^+^ increased with the addition of HNO_3_, possibly due to cation exchange between [HOOCmim]^+^ and H^+^ 9,36.

The traditional liquid-liquid extraction kinetics of [HOOCmim][NTf_2_] for the removal of UO_2_^2+^ has not previously been reported and was investigated at a constant temperature of 30 °C for comparison. Both *E*_U_ and distribution ratios (*D*_U_) increased rapidly and reached a plateau, with values exceeding 82.8 ± 3.2% and 3.4 ± 0.1, respectively, after 60 min (Fig. S4). This result indicates that the extraction equilibrium at 30 °C could be achieved in 60 min. Compared to traditional liquid-liquid extraction (Table 1), equilibration time for extraction is dramatically shortened through homogeneous extraction.

The mechanism of extraction using the [HOOCmim][NTf_2_]-H_2_O system is of great importance for its practical application, so it was studied by varying the H^+^ concentration of aqueous solution. As shown in Fig. 3, the partitioning of UO_2_^2+^ into the organic phase decreased rapidly as the H^+^ concentration was increased by addition of HNO_3_. Interestingly, the partitioning of UO_2_^2+^ into the organic phase increased after decreasing the H^+^ concentration by addition of NaOH solution. Nockemann *et al*.^19^ found that three carboxyl groups coordinated bidentately to the uranyl species in the crystal structure of [UO_2_([OOCmim])_3_]^2+^ complexes formed between [HOOCmim][NTf_2_] and UO_2_^2+^. Therefore, it can be proposed that deprotonation of the carboxyl groups is necessary for coordination of UO_2_^2+^. The deprotonation of [HOOCmim]^+^ can be inhibited by HNO_3_, but promoted by addition of NaOH. As a result, *E*_U_ decreases with addition of HNO_3_, but increases with addition of NaOH. Billard *et al*.^11^ proposed a cation exchange model between H^+^ and cationic species during extraction. Inhibition of the cation exchange mechanism by hydrogen ions has been proved in the literatures^11^,^36^,^41^,^42^. Therefore, the decrease of *E*_U_ into the organic phase is caused by both protonation of the carboxyl groups and inhibition of the cation exchange mechanism by hydrogen ions.

Based on the study of the extraction mechanism, the stripping of UO_2_^2+^ from the ionic liquid phase was performed by using nitric acid solution. The organic phase, containing UO_2_^2+^, was mixed with different concentrations of nitric acid solution. As illustrated in Fig. S5, the stripping of UO_2_^2+^ from carboxyl-functionalised task-specific ionic liquids was easily achieved using HNO_3_ solution, and nearly 97.3 ± 2.9% of the UO_2_^2+^ was stripped from the organic phase by 1 M HNO_3_. This approach provides a valuable method to strip the extracted UO_2_^2+^ and recycle carboxyl-functionalised task-specific ionic liquids.

The influence of metal ions on the extraction of UO_2_^2+^ was also assessed. As shown in Fig. 4, K^+^, Na^+^, Mg^2+^, Dy^3+^, La^3+^, and Eu^3+^ had little influence on the separation of UO_2_^2+^ from the aqueous phase, and the *E*_U_ remained at *ca*. 80%. These results suggest the potential for separation of UO_2_^2+^ in the presence of K^+^, Na^+^, Mg^2+^, Dy^3+^, La^3+^, and Eu^3+^. Furthermore, Eu^3+^ has been widely used as a representative of the trivalent lanthanides^43^. Accordingly, the extraction of Eu^3+^ was also studied under the same conditions to explore the selectivity of [HOOCmim][NTf_2_]-H_2_O. The results demonstrated that [HOOCmim][NTf_2_]-H_2_O had lower selectivity for Eu^3+^ (*E*_Eu_ = 42.1 ± 2.0%; *D*_Eu_ = 0.48 ± 0.02%) than UO_2_^2+^ (*E*_U_ = 83.2 ± 4.0%; *D*_U_ = 3.4 ± 0.2%), indicating the possibility for fast separation of UO_2_^2+^ from aqueous solution containing trivalent lanthanides.

The selectivity of [OOCmim] for UO_2_^2+^ and Eu^3+^ was further investigated using DFT calculations. Figure 5 shows the optimised structures of [OOCmim], [UO_2_([OOCmim])_3_]^2+^, and Eu([OOCmim])_4_]^3+^. Table 2 lists the changes in enthalpies (*H*_g_), entropies (*S*_g_), and binding energies (*G*_g_) for the metal-ligand complexation reactions in gas phase. As presented in Table 2, the gas-phase reaction enthalpies were relatively large, negative gas-phase binding energies that were significantly more negative than TΔ*S*_g_. The [OOCmim] showed high selectivity for Eu^3+^ (Δ*G*_*g*_ = −3005.2 kJ/mol) over UO_2_^2+^ (Δ*G*_*g*_ = −1610.9 kJ/mol) in the gas phase. In addition, considering solvent effects in [HOOCmim][NTf_2_] solution, the solvation structure was optimised in [HOOCmim][NTf_2_] and calculated by frequency analysis at the B3LYP/6-311 G(d,p)/RECP level of theory, based on the universal continuum solvation model of SMD. As shown in Table S2, the binding energies (Δ*G*_sol_) were much lower than the corresponding gas-phase binding energies. Interestingly, the difference in the Gibbs free energy for the complexation reactions in [HOOCmim][NTf_2_] proves that [OOCmim] has higher extractability for UO_2_^2+^ (Δ*G*_*sol*_ = −443.5 kJ/mol) compared to Eu^3+^ (Δ*G*_*sol*_ = −80.3 kJ/mol), which is remarkably consistent with the experimental results. Furthermore, the conformation of [UO_2_([OOCmim])_3_]^2+^ optimised in [HOOCmim][NTf_2_] agreed well with the reported crystal structure of [UO_2_([OOCmim])_3_]^2+^ (Fig. S6) in the literature^19^, which indicates that the solvation effect plays a significant role in the extraction of UO_2_^2+^. Consequently, the conformations of these species were affected by the solvation effect, leading to the clear changes of the Gibbs free energy for the complexation reactions and the selectivity of [OOCmim]. As shown in Table S3, for the formation of [UO_2_([OOCmim])_3_]^2+^ and Eu([OOCmim])_4_]^3+^, the changes of the Gibbs free energy in water were −152.2 and −47.5 kJ/mol, respectively, which are less negative compared to that of in [HOOCmim][NTf_2_]. The difference of the Gibbs free energy in different solvents suggests that these complexes are preferred in [HOOCmim][NTf_2_].

In conclusion, a new fast homogeneous system with [HOOCmim][NTf_2_] both as solvent and extractant is designed for the removal of UO_2_^2+^ from aqueous solution. The homogeneous phase of [HOOCmim][NTf_2_]-H_2_O system can be achieved at temperature higher than 75 °C, and 86.8% of UO_2_^2+^ was separated from the aqueous solution after vibrating for only 1 min. Compared to traditional liquid-liquid extraction, homogeneous extraction provides an extremely short equilibration time. Furthermore, nearly 97.3 ± 2.9% of UO_2_^2+^ can be stripped from organic phase by 1 M HNO_3_. K^+^, Na^+^, Mg^2+^, Dy^3+^, La^3+^, and Eu^3+^ have slight influence on the separation of UO_2_^2+^ from aqueous phase, and the *E*_U_ still remained at *ca*. 80%. [HOOCmim][NTf_2_]-H_2_O shows a high selectivity for UO_2_^2+^ rather than Eu^3+^, indicating the possibility for fast separation between UO_2_^2+^ and Eu^3+^. According to the results of DFT calculation, the solvent effect plays a significant role in the selectivity of [OOCmim]. The difference in the Gibbs free energy for the complexing reactions in [HOOCmim][NTf_2_] proves that [OOCmim] shows higher extractability for UO_2_^2+^ (Δ*G*_*sol*_ = −443.5 kJ/mol) than Eu^3+^ (Δ*G*_*sol*_ = −80.3 kJ/mol). Therefore, the fast homogeneous extraction system of [HOOCmim][NTf_2_]-H_2_O presents an opportunity for removal of UO_2_^2+^ in aqueous solution containing rare earth metal ions.

## Methods

### Materials

[HOOCmim][NTf_2_] (with a purity >99%) were purchased from Lanzhou Greenchem ILs, LICP, CAS, China (Lanzhou, China). UO_2_(NO_3_)_2_·6H_2_O was obtained from Beijer Chemapol Co. NaNO_3_, KNO_3_, Dy(NO_3_)_3_·6H_2_O, Eu(NO_3_)_3_·6H_2_O, and La(NO_3_)_3_·6H_2_O (Beijing chemical corp., >99%) were used to assess the influence of metal ions on the extraction of UO_2_^2+^. These compounds were used without further purification. All other solvents were analytical-grade reagent and used as received.

### Fast homogeneous extraction

Aqueous phase containing 2 mM UO_2_^2+^ was prepared by dissolving UO_2_(NO_3_)_2_·6H_2_O with deionized water in plastic container. 0.40 mL organic phase of [HOOCmim][NTf_2_] and 0.40 mL aqueous phase containing 2 mM UO_2_^2+^ were added into a tube. Then the tube was heated at 75 °C for 10 min, followed by vibrating for 1 min in a vibrating mixer. Hereafter, samples were kept in 60 °C and 30 °C thermostat for cooling. After that, samples were centrifuged for 5 min to ensure the complete separation of two phases. Then the aqueous solution was diluted *ca*. 40 times by deionized water, and the concentration of UO_2_^2+^ in the diluted aqueous solution was measured by Prodigy high dispersion inductively coupled plasma atomic emission spectrometer (ICP-AES) (Teledyne Leeman Labs, USA) at room temperature. Moreover, the influence of K^+^, Na^+^, Mg^2+^, Dy^3+^, La^3+^, and Eu^3+^ (2 mM) on the extraction of UO_2_^2+^ was assessed. The extraction of Eu^3+^ was also studied under the same condition for exploring the selectivity of [HOOCmim][NTf_2_]. The *E*_U_ and *D*_U_ are calculated as follows:


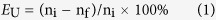



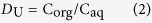


where n_i_ and n_f_ designate the initial and final amount of metal ions in the aqueous solution, respectively. C_org_ and C_aq_ represent the concentration of metal ions in the organic phase and the aqueous phase after extraction, respectively. All above experiments were carried out in plastic container, and all obtained values were in duplicate with uncertainty within 5%.

### Traditional liquid-liquid extraction

0.40 mL organic phase of [HOOCmim][NTf_2_] was mixed with 0.40 mL aqueous phase containing 2 mM UO_2_^2+^. The extraction experiments were oscillated with a rotating speed of 120 rpm in air bath at 30 °C. Afterwards, the samples were centrifuged for 5 min to ensure the complete separation of two phases. The *E*_U_ and *D*_U_ were calculated by using the same method as that of fast homogeneous extraction.

### Stripping experiment

After extraction, the organic phase containing UO_2_^2+^ was mixed with deionized water and different concentration of nitric acid solutions. The two phases were conducted in a vibrating mixer in order to make two phases completely contacted. The stripping efficiencies (*Es*) are calculated as follows:


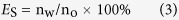


where n_o_ and n_w_ designate the initial amount of UO_2_^2+^ in the organic phase and the final amount of UO_2_^2+^ in the aqueous phase, respectively.

### The solubility of [HOOCmim]^+^ in water

#### ^1^H NMR

The solubility of [HOOCmim]^+^ in water (Fig. S2) during extraction were analysed by ^1^H NMR recorded on a Bruker AV-400 instrument.

### Theoretical calculations

Electron correlation effects are included by employing density functional theory (DFT) methods, which have shown that the main features of actinide complexes can be accurately reproduced at this level of theory^44^. Calculations were carried out with the Gaussian 09 program package using DFT at the B3LYP level^45^,^46^. For the U and Eu atoms, relativistic effects were considered with the quasirelativistic effective core potentials (RECPs) and the associated valence basis sets developed by the Stuttgart and Dresden groups^47^,^48^,^49^,^50^,^51^. The adopted large-core RECPs include 52 electrons^50^,^51^ and 60 electrons^47^,^48^,^52^ in the core for Eu(III) and U(VI) were used for geometry optimizations, respectively. The 6–311G(d,p) basis set was used for all carbon, hydrogen, oxygen, and nitrogen atoms. Geometry optimizations and electronic calculations for all of the species were carried out firstly in the gasphase at the B3LYP/6-311G(d,p)/RECP level. The enthalpies (*H*_g_), entropies (*S*_g_), and Gibbs free energies (*G*_g_) were calculated at the B3LYP/6-311G(d,p)/RECP level in the gas phase (298.15 K). For obtaining the enthalpies (*H*_sol_), entropies (*S*_sol_), and Gibbs free energies (*G*_sol_) of these species in solvents ([HOOCmim][NTf_2_] and water) at 298.15 K, these structures were optimised in solvents and calculated by frequency analysis at the B3LYP/6-311G(d,p)/RECP level of theory based on the universal continuum solvation model of SMD^53^, which was known to predict energies of solvation well^54^. The static dielectric constant at 66.4 determined by PCM-1A dielectric constant detector and refractive index at 1.4454 determined by Abbe refractometer were adopted for [HOOCmim][NTf_2_].

## Additional Information

**How to cite this article:** Ao, Y. *et al*. Fast selective homogeneous extraction of UO_2_^2+^ with carboxyl-functionalised task-specific ionic liquids. *Sci. Rep.* **7**, 44100; doi: 10.1038/srep44100 (2017).

**Publisher's note:** Springer Nature remains neutral with regard to jurisdictional claims in published maps and institutional affiliations.

## Supplementary Material

Supplementary Information

## Figures and Tables

**Figure 1:**
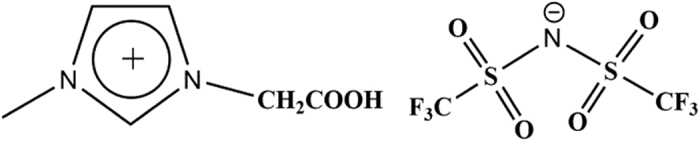
Chemical structure of [HOOCmim][NTf_2_].

**Figure 2:**
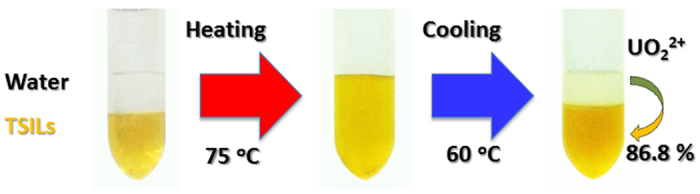
Fast homogeneous extraction of UO_2_^2+^ by [HOOCmim][NTf_2_].

**Figure 3:**
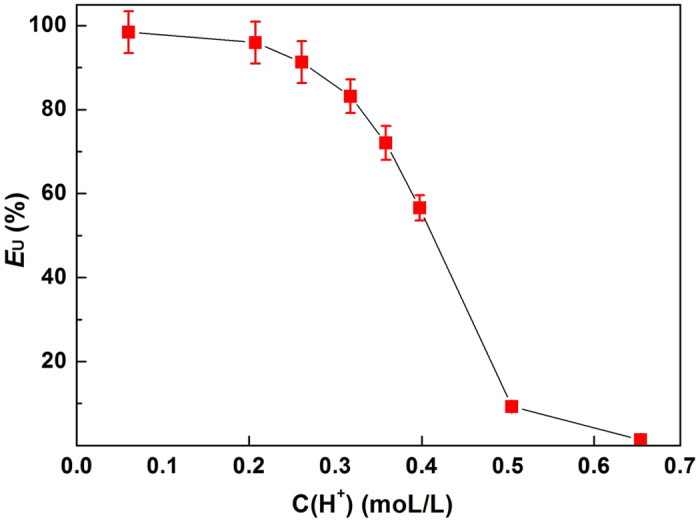
Influence of [H^+^] on the *E*_U_ of [HOOCmim][NTf_2_].

**Figure 4:**
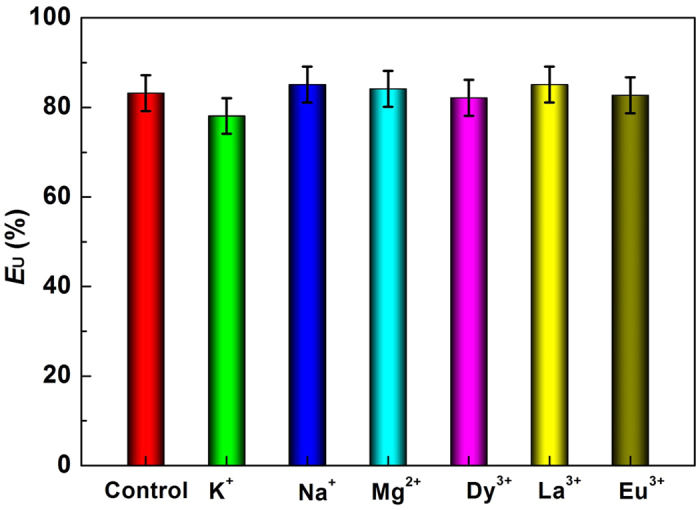
Influence of different metal ions on the extraction of UO_2_^2+^. ([UO_2_^2+^] = 2 mM; [M] = 2 mM, M = K^+^, Na^+^, Mg^2+^, Dy^3+^, La^3+^, Eu^3+^).

**Figure 5:**
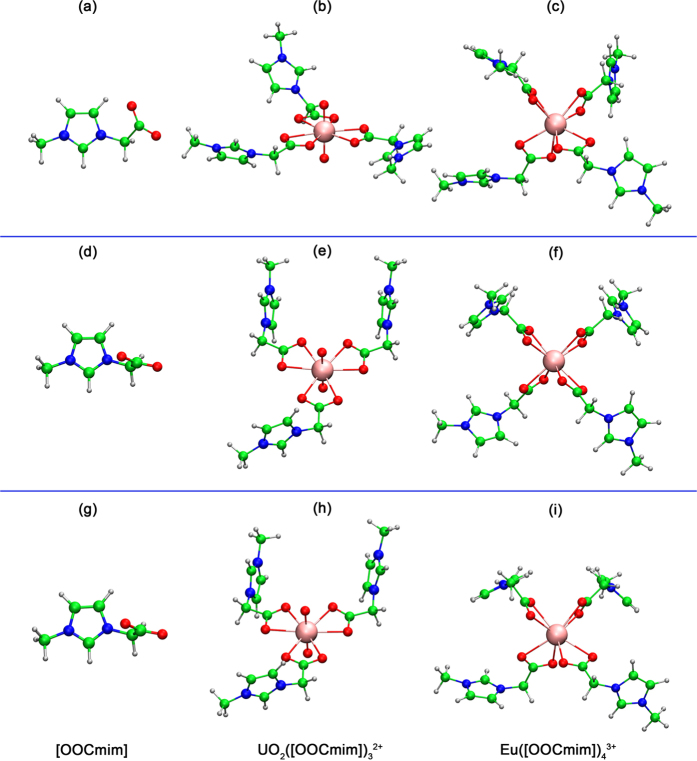
Optimised structures of [OOCmim], [UO_2_([OOCmim])_3_]^2+^, and Eu([OOCmim])_4_]^3+^. Green, white, red, blue, and light pink spheres represent C, H, O, N, and metal ion, respectively. ((**a**,**b**, and **c**) were obtained in gas phase. (**d**,**e**, and **f**) were obtained in [HOOCmim][NTf_2_]. (**g**,**h**, and **i**) were obtained in water).

**Table 1:** Traditional liquid-liquid extraction and homogeneous extraction of UO_2_^2+^ by various extraction systems.

Extraction System	Extraction Method	[UO_2_^2+^]	Solvent	Equilibrium Time	Distribution Ratios
TODGA/ILs^37^	Traditional Liquid - Liquid Extraction	1 mM	[C_6_mim][PF_6_]	120 min	*ca*. 21.5
TODGA/ILs^37^	Traditional Liquid - Liquid Extraction	1 mM	[C_8_mim][PF_6_]	120 min	*ca*. 4.3
TTA^38^	Traditional Liquid - Liquid Extraction	5 mM	[C_4_mim][NTf_2_]	30 min	53.2
TBP^38^	Traditional Liquid - Liquid Extraction	5 mM	[C_4_mim][NTf_2_]	30 min	15.7
CMPO^39^	Traditional Liquid - Liquid Extraction	10 mM	[C_4_mim][NTf_2_]	24 h	2.6
[HOOCmim][NTf_2_]	Traditional Liquid - Liquid Extraction	2 mM	—	60 min	3.44
[HOOCmim][NTf_2_]	Homogeneous Extraction	2 mM	—	1 min	3.46
[AOmim][NTf_2_]^40^	Homogeneous Extraction	0.005 mCi	—	—	7.9
[Hbet][NTf_2_]^33^	Homogeneous Extraction	20 mM	—	7 min	*ca*. 1.7

TODGA: *N,N,N’,N’*-tetraoctyldiglycolamide; [C_6_mim][PF_6_]: 1-hexyl-3-methylimidazolium hexafluorophosphate; [C_8_mim][PF_6_]: 1-octyl -3-methylimidazolium hexafluorophosphate; [C_4_mim][NTf_2_]: 1-Butyl-3-methylimidazoliumbis(trifluoromethylsulfonyl)imide; TBP: Tributylphosphate; TTA: Thenoyltrifluoroacetone; CMPO: Octylphenyl-*N,N*-diisobutylcarbamoylmethylphosphine oxide; [AOmim][NTf2]: Amidoxime functionalized alkylation of 1-methylimidazole bis(trifluoromethane)sulfonamide; [Hbet][NTf_2_]: Betainium Bis(trifluoromethylsulfonyl)imide.

**Table 2:** The changes in enthalpy, entropy, and binding energies (298.15 K, kJ/mol) for the complexes between [OOCmim] and metal ions obtained separately in gas phase, [HOOCmim][NTf_2_], and water by the B3LYP/6-311G(d,p)/RECP level.

(**a**) **Complexation in gas phase**	**Δ*****H***_***g***_	***T*****Δ*****S***_***g***_	**Δ*****G***_***g***_
UO_2_^2+^+3[OOCmim] → [UO_2_([OOCmim])_3_]^2+^	−1745.7	−134.8	−1610.9
Eu^3+^+4[OOCmim] → Eu([OOCmim])_4_]^3+^	−3184.5	−179.3	−3005.2
(**b**) **Complexation in** [**HOOCmim**][**NTf**_**2**_]	**Δ*****H***_***sol***_	***T*****Δ*****S***_***sol***_	**Δ*****G***_***sol***_
UO_2_^2+^+3[OOCmim] → [UO_2_([OOCmim])_3_]^2+^	−597.7	−154.2	−443.5
Eu^3+^+4[OOCmim] → Eu([OOCmim])_4_]^3+^	−272.1	−191.8	−80.3
(**c**) **Complexation in water**	**Δ*****H***_***sol***_	***T*****Δ*****S***_***sol***_	**Δ*****G***_***sol***_
UO_2_^2+^+3[OOCmim] → [UO_2_([OOCmim])_3_]^2+^	−314.7	−162.5	−152.2
Eu^3+^+4[OOCmim] → Eu([OOCmim])_4_]^3+^	−237.3	−189.8	−47.5
