# Supplementary material for: Fast selective homogeneous extraction of UO22+ with carboxyl-functionalised task-specific ionic liquids
Source: Sci Rep. 2017 Mar 14;7:44100. doi: 10.1038/srep44100 (PMC5349554; doi:10.1038/srep44100)
Supplement: Supplementary Information [file srep44100-s1.pdf]

Supporting Information

Fast selective homogeneous extraction of UO_2^{2+} with carboxyl-functionalised task-specific ionic liquids †

Yinyong Ao [1,2*], Jian Chen [1,2], Min Xu [2], Jing Peng [2], Wei Huang [1], Jiuqiang Li [2], and Maolin Zhai [2]*

[1] Institute of Nuclear Physics and Chemistry, China Academy of Engineering Physics, Mianyang 621900, P. R. China

[2] Beijing National Laboratory for Molecular Sciences (BNLMS), Department of Applied Chemistry, College of Chemistry and Molecular Engineering, Peking University, Beijing 100871, P. R. China

* Corresponding authors: Tel/Fax: +86-0816-2482203, E-mail: aoyinyong@126.com; Tel/Fax: +86-10-62753794; E-mail: mlzhai@pku.edu.cn

Page No.	Contents
**S2**	Fig S1. Relationship between the rate of $[HOOCmim]^+$ from organic phase to aqueous phase and phase ratio of V_{TSILs}/V_{H2O}.
**S3**	Fig S2. NMR spectra of $[HOOCmim]^+$ varying with different equilibrium time.
**S3**	Fig S3. Influence of equilibrium time on the solubility of $[HOOCmim]^+$ in water.
**S4**	Fig S4. Influence of equilibrium time on E_U and D_U of $[HOOCmim][NTf_2]$ system.
**S5**	Fig S5. The stripping of UO_2^{2+} from organic phase by HNO_3.
**S6**	Table S1. The enthalpy, entropy, and binding energies (298.15 K) for [OOCmim], metal ions and complexes obtained in gas phase by DFT method at B3LYP/6-311G(d,p)/RECP level.
**S6**	Table S2. The enthalpy, entropy, and binding energies (298.15 K) for [OOCmim], metal ions and complexes obtained in $[HOOCmim][NTf_2]$ by DFT method at B3LYP/6-311G(d,p)/RECP level.
**S6**	Table S3. The enthalpy, entropy, and binding energies (298.15 K) for [OOCmim], metal ions and complexes obtained in water by DFT method at B3LYP/6-311G(d,p)/RECP level.
**S7**	Fig S6. Optimized structures of $[UO_2([OOCmim])_3]^{2+}$ in gas phase (a) and in $[HOOCmim][NTf_2]$ (b), and the crystal structure (c) of $[UO_2([OOCmim])_3]^{2+}$.

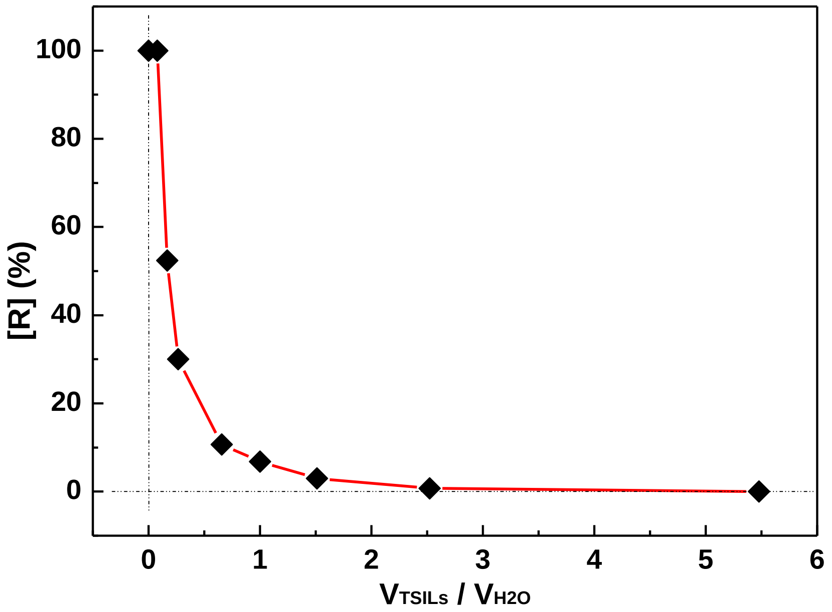

Fig S1. Relationship between the rate of [HOOCmim]$^+$ from organic phase to aqueous phase and phase ratio of V_{TSILs}/V_{H2O}. V_{TSILs} and V_{H2O} represent the initial volume of [HOOCmim][NTf_2] and water, respectively.

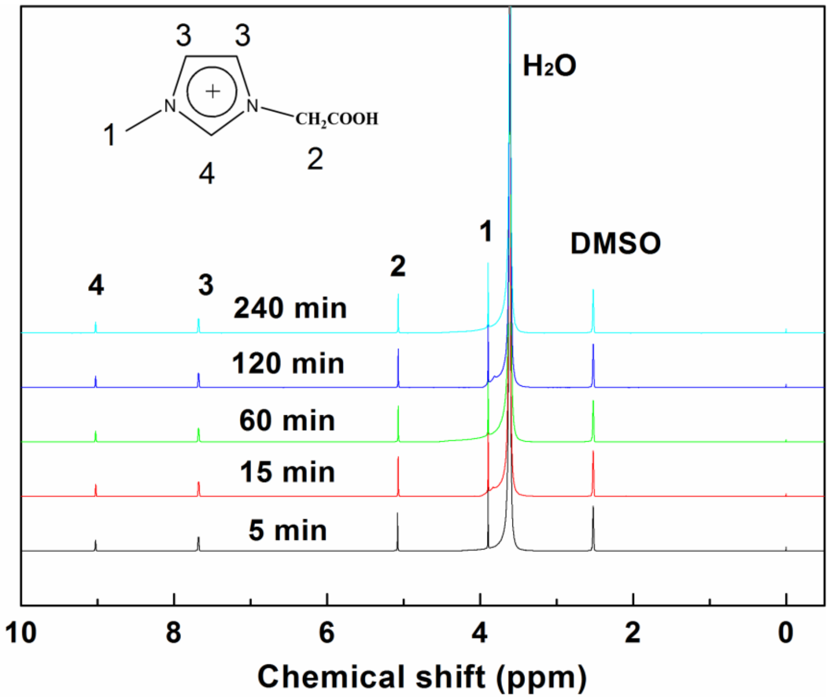

Fig S2. NMR spectra of [HOOCmim]$^+$ varying with different equilibrium time.

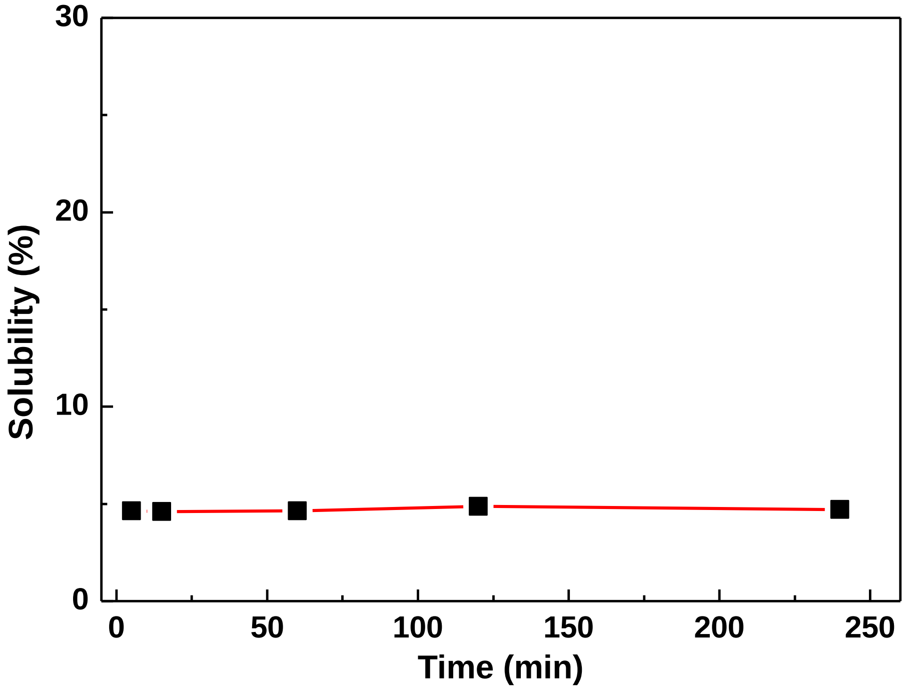

Fig S3. Influence of equilibrium time on the solubility of [HOOCmim]$^+$ in water.

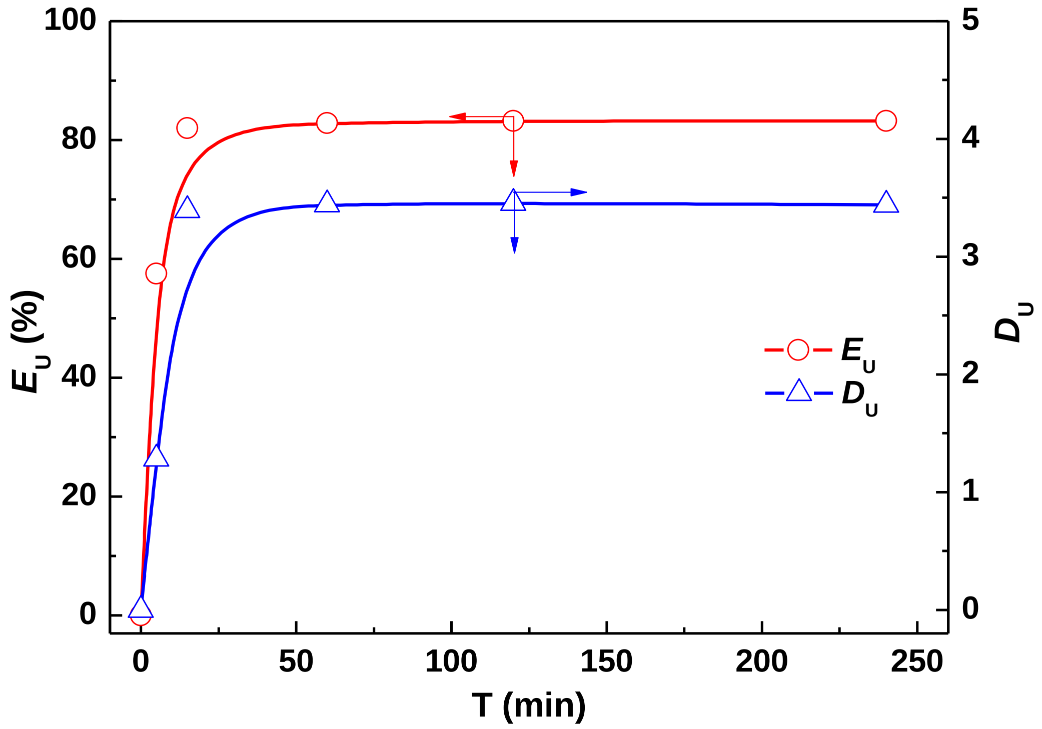

Fig S4. Influence of equilibrium time on E_U and D_U of [HOOCmim][NTf_2] system.

([UO_2^{2+}] = 2 mM; Temperature = 30 ^oC)

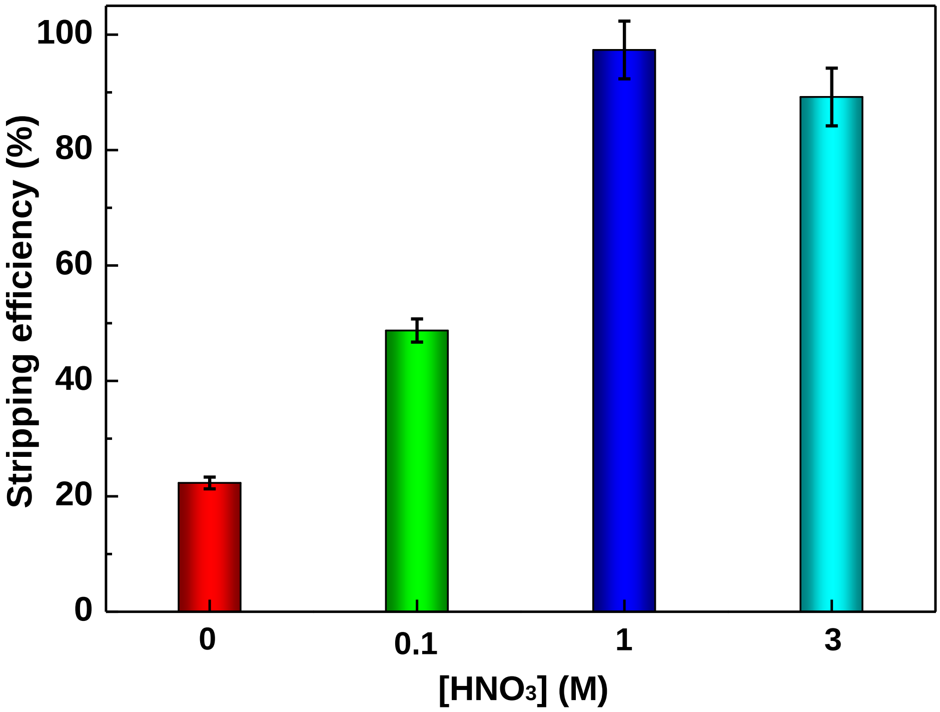

Fig S5. The stripping of UO_2^{2+} from organic phase by HNO_3.

Table S1. The enthalpy, entropy, and binding energies (298.15 K) for [OOCmim], metal ions and complexes obtained in gas phase by DFT method at B3LYP/6-311G(d,p)/RECP level.

**Species**	$\boldsymbol{H_g}$ **(a.u.)**	$\boldsymbol{G_g}$ **(a.u.)**	**T$\boldsymbol{S_g}$ (kJ/mol)**
**[OOCmim]**	-493.34752	-493.39443	123.16
$\mathbf{UO_2^{2+}}$	-626.78514	-626.81026	65.94
$\mathbf{[UO_2([OOCmim])_3]^{2+}}$	-2107.49260	-2107.60712	300.68
$\mathbf{Eu^{3+}}$	-33.81676	-33.83623	51.13
$\mathbf{Eu([OOCmim])_4]^{3+}}$	-2008.41973	-2008.55859	364.57

Basis set: 6-311G(d,p) for C, H, O, N; ECP60MWB for U(VI); ECP52MWB for Eu(III).

Table S2. The enthalpy, entropy, and binding energies (298.15 K) for [OOCmim], metal ions and complexes obtained in [HOOCmim][NTf_2] by DFT method at B3LYP/6-311G(d,p)/RECP level.

**Species**	$\boldsymbol{H_{sol}}$ **(a.u.)**	$\boldsymbol{G_{sol}}$ **(a.u.)**	**T$\boldsymbol{S_{sol}}$ (kJ/mol)**
**[OOCmim]**	-493.40637	-493.45248	121.07
$\mathbf{UO_2^{2+}}$	-627.33115	-627.36117	78.80
$\mathbf{[UO_2([OOCmim])_3]^{2+}}$	-2107.77790	-2107.88755	287.90
$\mathbf{Eu^{3+}}$	-35.16335	-35.18282	51.13
$\mathbf{Eu([OOCmim])_4]^{3+}}$	-2008.89247	-2009.02333	343.57

Basis set: 6-311G(d,p) for C, H, O, N; ECP60MWB for U(VI); ECP52MWB for Eu(III).

Table S3. The enthalpy, entropy, and binding energies (298.15 K) for [OOCmim], metal ions and complexes obtained in water by DFT method at B3LYP/6-311G(d,p)/RECP level.

**Species**	$\boldsymbol{H_{sol}}$ **(a.u.)**	$\boldsymbol{G_{sol}}$ **(a.u.)**	**T$\boldsymbol{S_{sol}}$ (kJ/mol)**
**[OOCmim]**	-493.40756	-493.45297	119.21
$\mathbf{UO_2^{2+}}$	-627.41042	-627.44090	80.03
$\mathbf{[UO_2([OOCmim])_3]^{2+}}$	-2107.75297	-2107.85777	275.16
$\mathbf{Eu^{3+}}$	-35.16649	-35.18597	51.13
$\mathbf{Eu([OOCmim])_4]^{3+}}$	-2008.88715	-2009.01592	338.09

Basis set: 6-311G(d,p) for C, H, O, N; ECP60MWB for U(VI); ECP52MWB for Eu(III).

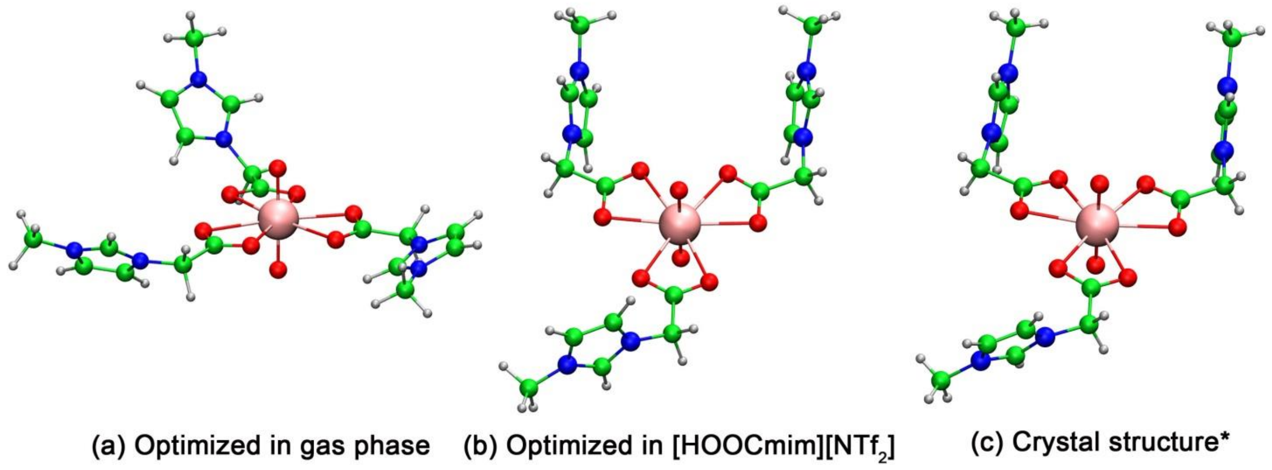

Fig S6. Optimized structures of $[UO_2([OOCmim])_3]^{2+}$ in gas phase (a) and in $[HOOCmim][NTf_2]$ (b), and the crystal structure of $[UO_2([OOCmim])_3]^{2+}$ (c). Green, white, red, blue, and light pink spheres represent C, H, O, N, and metal ions, respectively.

*P. Nockemann, R. Van Deun, B. Thijs, D. Huys, E. Vanecht, K. Van Hecke, L. Van Meervelt and K. Binnemans, *Inorg. Chem.*, 2010, **49**, 3351-3360.
